# Aberrant Glycosylation in the Left Ventricle and Plasma of Rats with Cardiac Hypertrophy and Heart Failure

**DOI:** 10.1371/journal.pone.0150210

**Published:** 2016-06-09

**Authors:** Chiaki Nagai-Okatani, Naoto Minamino

**Affiliations:** Department of Molecular Pharmacology, National Cerebral and Cardiovascular Center Research Institute, Suita, Osaka, Japan; University at Buffalo, UNITED STATES

## Abstract

Targeted proteomics focusing on post-translational modifications, including glycosylation, is a useful strategy for discovering novel biomarkers. To apply this strategy effectively to cardiac hypertrophy and resultant heart failure, we aimed to characterize glycosylation profiles in the left ventricle and plasma of rats with cardiac hypertrophy. Dahl salt-sensitive hypertensive rats, a model of hypertension-induced cardiac hypertrophy, were fed a high-salt (8% NaCl) diet starting at 6 weeks. As a result, they exhibited cardiac hypertrophy at 12 weeks and partially impaired cardiac function at 16 weeks compared with control rats fed a low-salt (0.3% NaCl) diet. Gene expression analysis revealed significant changes in the expression of genes encoding glycosyltransferases and glycosidases. Glycoproteome profiling using lectin microarrays indicated upregulation of mucin-type *O*-glycosylation, especially disialyl-T, and downregulation of core fucosylation on *N*-glycans, detected by specific interactions with *Amaranthus caudatus* and *Aspergillus oryzae* lectins, respectively. Upregulation of plasma α-l-fucosidase activity was identified as a biomarker candidate for cardiac hypertrophy, which is expected to support the existing marker, atrial natriuretic peptide and its related peptides. Proteomic analysis identified cysteine and glycine-rich protein 3, a master regulator of cardiac muscle function, as an *O*-glycosylated protein with altered glycosylation in the rats with cardiac hypertrophy, suggesting that alternations in *O*-glycosylation affect its oligomerization and function. In conclusion, our data provide evidence of significant changes in glycosylation pattern, specifically mucin-type *O*-glycosylation and core defucosylation, in the pathogenesis of cardiac hypertrophy and heart failure, suggesting that they are potential biomarkers for these diseases.

## Introduction

Heart failure (HF), a leading cause of morbidity and mortality worldwide, is a cardiac dysfunction syndrome, induced by several factors related to cardiac volume and/or pressure overload. Natriuretic peptides, including atrial natriuretic peptide (ANP) and B-type natriuretic peptide (BNP), are powerful vasodilators regulating blood pressure and bodily fluid volume, and are secreted by cardiomyocytes in response to stretch stress [1]. Therefore, these biologically active peptides are used for diagnosis and prognosis of HF [1]. Particularly, BNP and the N-terminal intervening peptide of BNP precursor (NT-proBNP) are gold standard biomarkers of HF in humans [2], although ANP and the N-terminal fragment of its precursor (NT-proANP) play a predominant role in animal models. As HF pathophysiology is multifactorial and complicated, combined profiling of multiple biomarkers indicating independent biological processes is important for accurate evaluation and management of patients with HF [3]. Because of a strong demand for new biomarkers, extensive research has been conducted to provide biomarker candidates that surpass or complement natriuretic peptides in HF diagnosis; however, only few have met the criteria for clinical use [4].

Most eukaryotic proteins are subjected to post-translational modifications that regulate their functional properties, including activity, localization, turnover, and interactions with other proteins [5]. Qualitative and quantitative changes in post-translational modifications affect cellular homeostasis and often underlie pathophysiological processes; therefore, analysis of protein post-translational modifications is widely utilized for unbiased biomarker screening [5–7]. Glycosylation is the most common and frequent protein post-translational modification, and many glycoproteins are shed or secreted from cells and are in circulation [8]. Therefore, glycosylation profiling of blood proteins is recognized as a promising approach to the discovery of novel circulating biomarkers for clinical use.

Aberrant glycosylation of several proteins, including those of the cardiac t-tubular systems, occurs during the progression of cardiac hypertrophy and HF [9–11], indicating that HF development is associated with disturbances in glycosylation-regulating machinery. Nevertheless, glycoproteomic approaches have not been fully applied to the investigation of the molecular pathophysiology underlying cardiac hypertrophy and resultant HF, mostly because of insufficient knowledge of the mechanisms involved in cellular glycoproteome dynamics over the course of disease development.

Here, we characterized alterations in the protein glycosylation machinery and the resultant aberrant glycoproteome of Dahl salt-sensitive (DS) rats, a model of hypertension-induced cardiac hypertrophy and HF [12]. We present a biomarker candidate for cardiac hypertrophy with characteristics that differ from those of ANP, and propose a possible relationship between altered glycosylation and cardiac dysfunction.

## Materials and Methods

### Animals

All animals were maintained at an animal facility accredited by the Center for Accreditation of Laboratory Animal Care and Use, Japan Health Sciences Foundation. Protocols for animal experiments were approved by the Animal Care and Use Committee of National Cerebral and Cardiovascular Center, which conforms to the US National Institute of Health Guide for the Care and Use of Laboratory Animals. Four-week-old male DS rats (Japan SLC, Shizuoka, Japan) were fed a control low-salt (LS) diet containing 0.3% NaCl (Oriental Yeast, Tokyo, Japan) for 2 weeks. Rats were then randomly assigned to groups fed either a LS or high-salt (HS) diet containing 8% NaCl (Oriental Yeast). Animals were housed three per cage in a room maintained at 23–25°C with a 12-h light:12-h dark cycle; food and water were provided *ad libitum*.

### Tissue and plasma collection

At 12 and 16 weeks, rats were anesthetized with an intraperitoneal injection of pentobarbital (50 mg/kg body weight). Whole blood was collected from cardiac ventricles into a chilled tube containing 1/10 volume of EDTA-2Na (15 mg/mL) and aprotinin (5,000 kallikrein inhibitor units/mL). After centrifugation at 1,000 × *g* for 15 min at 4°C, plasma was collected and stored at -80°C until use. Immediately after blood collection, the anesthetized rats were perfused from the left ventricle (LV) with saline containing heparin (5 units/mL) for 15 min. Tissues were collected from the anesthetized rats, quickly diced, frozen in liquid nitrogen, and stored at -80°C until use. The LV tissue fragments were collected from the center region of the left ventricular (LV) free wall of each rat to minimize the experimental variations. Recombinant human cysteine and glycine-rich protein 3 (CSRP3) expressed in *E*. *coli* and HEK293 cells were purchased from Abcam (approximately 21 kDa, #ab40097; Cambridge, MA) and OriGene (approximately 25 kDa, #TP325217; Rockville, MD), respectively.

### Measurement of blood pressure

Systolic blood pressure was measured between 10:00 and 12:00 am by the tail-cuff method without anesthesia, using a programmable sphygmomanometer (BP-98A; Softron, Tokyo, Japan).

### Echocardiography

Rats lightly anesthetized by isoflurane inhalation (3%) via nose cone were evaluated by transthoracic echocardiography (Vevo 2100 imaging system; VisualSonics, Toronto, Canada) equipped with a 30-MHz transducer (MS-440). Digital images obtained from the M-mode echocardiography were analyzed using the Vevo 2100 workstation software to measure LV cavity size and wall thickness.

### Gene expression analysis

Total RNA was extracted from tissues and treated with DNase by using the RNeasy Mini kit (Qiagen, Valencia, CA). For evaluating the expression levels of 85 genes (S1 Table) using the RT^2^ Profiler PCR Array Rat Glycosylation Kit (PARN-046Z, SABiosciences, Frederick, MD), total RNA (1 μg) was reverse-transcribed to cDNA by using the RT^2^ First Strand Kit (Qiagen) and used for the 96-well plate-formatted array. The quantitative polymerase chain reaction (qPCR) array was performed according to the manufacturer’s protocol by using a LightCycler 480 System (Roche, Indianapolis, IN). Expression analysis was performed by using the manufacturer’s online analysis tool, and the expression levels of the glycogenes (glycosyltransferase, glycosidase, and other glycosylation-related genes) in the LV were normalized to those of the following three housekeeping genes: TATA box-binding protein (*Tbp*; UniGene accession no. Rn.22712), ribosomal protein S18 (*Rps18*; Rn.42766), and hypoxanthine phosphoribosyltransferase 1 (*Hprt1*; Rn.47), which were selected from 12 genes preset in the Rat Housekeeping Gene Primer Set (Takara Bio, Shiga, Japan). For qPCR with gene-specific primer pairs (S2 Table), cDNA was synthesized using SuperScript III reverse transcriptase (Life Technologies, Carlsbad, CA), and qPCR was performed using SYBR Premix Ex Taq II (Takara Bio) and the LightCycler 480 System. The cycling conditions were as follows: denaturation at 95°C for 30 s; 45 cycles at 95°C for 10 s and at 60°C for 20 s, followed by melting curve analysis. *Tbp* and β-actin (*Actb*; Rn.94978) were used as reference genes for the LV and liver, respectively. Relative expression of target genes normalized to the reference gene (S2 Table) was quantified using the LightCycler 480 software (Roche) based on the E-method analysis.

### Lectin microarray

LV extract for the lectin microarray was prepared by homogenization of LV tissues in lysis buffer (10 mM phosphate buffer, pH 7.4, 140 mM NaCl, 2.7 mM KCl, 1% Triton X-100) supplemented with a protease inhibitor cocktail (#P8340, Sigma-Aldrich, St. Louis, MO) and centrifugation at 14,000 × *g* for 5 min at 4°C; the supernatant was used for analysis. Plasma was passed through a Seppro rat spin column (Sigma-Aldrich) to remove seven highly abundant proteins (albumin, α_1_-antitrypsin, transferrin, fibrinogen, immunoglobulins G and M, and haptoglobin). Lectin microarray analysis of the LV extracts and the depleted plasma was performed as described elsewhere [13–15]. Briefly, proteins labeled with Cy3 mono-reactive dye (GE Healthcare, Piscataway, NJ) were quantified using the BCA assay (Thermo Fisher Scientific, Rockford, IL) and applied to a LecChip (GlycoTechnica, Yokohama, Japan), which contained 45 immobilized lectins (S3 and S4 Tables) on a slide glass. The slide was scanned using an evanescent-field fluorescence scanner (GlycoStation; GlycoTechnica) to detect Cy3-fluorescence of lectin-bound glycoproteins, and resulting digital images were analyzed with the GlycoStation Tools Pro ver. 1.5 (GlycoTechnica) by applying a gain-merging technique [13] and mean normalization [14]. Note that undetectable signal intensity do not necessarily indicate the absence of glycans bound to the lectin of interest in the sample, because this analysis used data obtained in an optimized condition without signal saturation for all the lectins on the array chip.

### Western blot and lectin blot analyses

LV extracts for western blot and lectin blot analyses were prepared using T-PER lysis buffer (Thermo Fisher Scientific) supplemented with protease inhibitor cocktail, according to the manufacturer’s protocol. Fractionation of sialidase-treated LV extracts was performed by ammonium sulfate precipitation using a 2-D Fractionation Kit (GE Healthcare), according to the manufacturer’s protocol after the addition of lysis buffer to the sialidase-treated LV extracts. Immunoprecipitation of rat and human CSRP3 were conducted using the Dynabeads Protein G Immunoprecipitation Kit (Life Technologies), according to the manufacturer’s protocol, with a mouse anti-CSRP3 antibody (B-4: #sc-393599, Santa Cruz Biotechnology, Dallas, TX); 2 μg of the antibody and 50 μl of the magnetic beads were used for immunoprecipitation from 1 mg of LV extracts. Protein concentration was estimated with the 660 nm Protein Assay Reagent (Thermo Fisher Scientific) by using bovine serum albumin as a standard. Ten micrograms of total protein were dissolved in sample buffer containing 2-mercaptoethanol, heated, separated by sodium dodecyl sulfate-polyacrylamide gel electrophoresis (SDS-PAGE), and transferred onto polyvinylidene difluoride membranes (Hybond-P; GE Healthcare). For analysis in unheated and non-reducing conditions, protein samples were dissolved in sample buffer without 2-mercaptoethanol without heating.

For western blotting, the membranes were blocked with 2% ECL Prime Blocking Reagent (GE Healthcare) in TBS-T buffer (25 mM Tris-HCl, 150 mM NaCl, 0.1% Tween 20, pH 7.4) at room temperature for 1 h, incubated overnight at 4°C with primary antibodies, and then incubated with appropriate horseradish peroxidase-conjugated secondary antibodies at room temperature for 1 h. Primary antibodies against the following proteins were used: rat β-actin (ACTB, 1:10,000; #109639, GeneTex, San Antonio, TX), glycoprotein-*N*-acetylgalactosamine 3-β-galactosyltransferase (C1GALT1,1:1,000; #46062, GeneTex), C1GALT1 molecular chaperone (C1GALT1C1, 1:1,000; #115877, GeneTex), α-l-fucosidase (FUCA1, 1:1,000; #AV54293, Sigma-Aldrich), *N*-acetylgalactosaminyltransferase 1 (GALNT1, 1:300; #sc-68492, Santa Cruz Biotechnology), GALNT2 (1:2,000; #104070, GeneTex), GALNT7 (1:10,000; #106068, GeneTex), glycerardehyde-3-phosphate dehydrogenase (GAPDH, 1:1,000; #2118, Cell Signaling Technology, Beverly, MA), β-galactosidase (GLB1, 1:3,000; #104360, GeneTex), *O*-GlcNAc hydrolase (OGA, 1:500; #14711-1-AP, Proteintech, Chicago, IL), *O*-GlcNAc transferase (OGT, 1:3,000; #109939, GeneTex), and CSRP3 (1: 2000; #88505, GeneTex). Horseradish peroxidase-conjugated secondary antibodies were goat anti-rabbit IgG (H+L) (1:2,000, #7074; Cell Signaling Technology) and rabbit anti-goat IgG (H+L) (1:2,000, #305-035-003; Jackson ImmunoResearch, West Grove, PA). The primary and secondary antibodies were diluted with Immuno Shot Reagents 1 and 2 (Cosmo Bio, Tokyo, Japan), respectively.

Lectin blot analysis was performed basically in accordance with the protocol reported elsewhere [16]. Briefly, the membranes were blocked at room temperature for 1 h with 1 × Carbo-Free blocking solution (Vector Laboratories, Burlingame, CA) containing 0.1% Tween 20, incubated overnight at 4°C with 1 μg/mL fluorescein isothiocyanate-conjugated *Amaranthus caudatus* lectin (ACA; Vector Laboratories) or *Aspergillus oryzae* lectin (AOL; Tokyo Chemical Industry, Tokyo, Japan) diluted with blocking solution, and then incubated at room temperature for 1 h with rabbit anti-fluorescein isothiocyanate Fab′ (1:1,000; Dako, Carpinteria, CA) diluted with blocking solution. At least three independent experiments were performed after optimizing the experimental conditions of each lectin blotting.

Chemiluminescent signals were detected by Chemi-Lumi One Super or Ultra (Nacalai Tesque, Kyoto, Japan), using a LAS 4000 system (Fujifilm, Tokyo, Japan), and densitometry analysis was performed using the ImageQuant software (GE Healthcare). Densitometry data were normalized to the signal intensity of glycerardehyde-3-phosphate dehydrogenase for western blotting and to total proteins stained with SYPRO Ruby for lectin blotting.

### Two-dimensional electrophoresis analysis and protein identification

For two-dimensional electrophoresis analysis, sialidase-treated LV extracts were purified with a ReadyPrep 2-D cleanup kit (Bio-Rad, Hercules, CA) and then dissolved in a rehydration buffer (7 M urea, 2 M thiourea, 4% w/v CHAPS, 0.5% v/v IPG buffer [pH 6–11; GE Healthcare], and 15 mg/ml DeStreak Reagent). For the first dimension separation, isoelectric focusing was carried out using the Ettan IPGphor 3 IEF System (GE Healthcare) with Immobiline DryStrip (13 cm, pH 6–11; GE Healthcare) at 50 μA/strip. A total amount of 50 μg and 200 μg of protein was loaded onto each strip for profiling analysis and identification of protein spots, respectively. The program for the first dimension was as follows: 500 V for 1 h, 1000 V gradient for 1 h, 8000 V gradient for 2.5 h, and 8000 V for 0.5 h. The strips were then equilibrated with equilibration buffer (50 mM Tris-HCl, pH 8.8, containing 6 M urea, 30% w/v glycerol, 2% w/v SDS, and a trace of bromophenol blue) twice for 15 min; dithiothreitol (0.5% w/v) was added to the first, and iodoacetamine (4.5% w/v) to the second equilibration step. Electrophoresis in the second dimension was performed using a 10–20% gradient gel (Perfect NT Gel S; DRC, Tokyo, Japan). After the run, the gels were stained with Lumitein (Biotium, Hayward, CA). Protein spots of interest were excised using a gel picker (FluoroPhoreStar 3000; Anatech, Tokyo, Japan) and digested with sequencing-grade modified porcine trypsin (Promega, Madison, WI) and ProteaseMax Surfactant (Promega), according to the manufacturers’s protocols. Subsequent protein identification was carried out with a 5800 matrix-assisted laser desorption ionization -time of flight mass spectrometer (AB Sciex, Framingham, MA) on a reflector mode. The product ion spectra generated by MS/MS analysis were searched against the Swiss-Prot database for exact matches using the MASCOT MS/MS ion search program (http://www.matrixscience.com).

### Enzyme activity assays

For the enzyme activity assays, tissue extracts were prepared using T-PER lysis buffer, as described above. T-synthase activity was measured as described elsewhere [17]. Briefly, 40 μL of reaction solution containing 1.25 mM 4-methylumbelliferyl (4MU)- 2-acetamido-2-deoxy-α-d-galactopyranoside (Carbosynth, Berkshire, UK), 0.625 mM uridine diphosphate-d-galactose (Millipore, Bedford, MA), 20 mM MnCl_2_, 0.2% Triton X-100, and 20,000 U/mL *O*-glycosidase (New England Biolabs, Ipswich, MA) in 25 mM MES buffer (pH 6.8) was mixed with 10 μL of the tissue extract in a 96-well FluoroNunc MaxiSorp black microtiter plate (Nunc, Naperville, IL). After incubation at 37°C for 1 h, 100 μL of stop solution (1 M glycine-NaOH, pH 10.0) was added to each well.

α-l-fucosidase (AFU) activity was measured as described elsewhere [18]. Briefly, 40 μL of reaction solution containing 1 mM of a synthetic substrate 4-MU-α-l-fucopyranoside (Glycosynth, Cheshire, UK) in 0.1 M citrate/phosphate buffer (pH 5.0) was mixed with 10 μL of tissue extract diluted 1:5–1:25 (v/v) with citrate/phosphate buffer (pH 5.0) and incubated at 37°C for 1 h. The reaction was stopped as described above and fluorescence (excitation 355 nm, emission 460 nm) was measured using a fluorescence microplate reader (SpectraMax M2e; Molecular Devices, Sunnyvale, CA). The amount of 4-MU produced by α-l-fucosidase was determined based on the standard curve of 4-MU (Sigma-Aldrich). All the experiments were performed in duplicate. One unit of enzyme activity was defined as the amount of enzyme generating 1 nmol 4-MU per min at 37°C.

### Measurement of plasma levels of N-terminal fragments of natriuretic peptides

Plasma NT-proANP and NT-proBNP levels in the rats were measured using double-antibody radioimmunoassays developed in our laboratory, as reported previously [19,20]. For the rat NT-proANP radioimmunoassay, we used rabbit polyclonal antiserum #593–12 raised against human proANP[1–31]+C (amino acid sequence: NPMYNAVSNADLMDFKNLLDHLEEKMPLEDEC) and keyhole limpet hemocyanin conjugate at a final dilution of 1:36,000 with a setting of rat proANP[1–31]+Y (amino acid sequence: NPVYSAVSNTDLMDFKNLLDHLEEKMPVEDEY) as a standard and its ^125^I-labeled peptide as a tracer. Half maximum inhibition of tracer binding in this RIA was observed at 20 fmol/tube. Rat NT-proBNP RIA was performed with rabbit polyclonal antiserum #601–8 raised against rat proBNP[1–28]+CY (amino acid sequence: HPLGSPSQSPEQSTMQKLLELIREKSEECY) and keyhole limpet hemocyanin conjugate at a final dilution of 1:330,000, and rat proBNP[1–28]+SY (HPLGSPSQSPEQSTMQKLLELIREKSEESY) and its ^125^I-labeled peptide as a standard and a tracer, respectively. Half maximum inhibition of tracer binding in the NT-proBNP RIA was 10 fmol/tube. Plasma NT-proANP and NT-proBNP levels were measured by the double antibody radioimmunoassay method as reported previously for ANP measurement.[19,21] Briefly, a mixture of 100 μl each of sample and antibody solutions of RIA buffer (50 mM sodium phosphate [pH 7.4] containing 80 mM NaCl, 25 mM EDTA, 0.05% NaN_3_, 0.5% bovine serum albumin, and 0.5% Triton X-100) was preincubated at 4°C overnight and then incubated with 100 μl of tracer solution in the RIA buffer containing dextran T-40 at the final concentration of 1.0%, at 4°C for 40 h. Normal rabbit serum and goat anti-rabbit IgG antiserum (Shibayagi, Gunma, Japan) diluted with RIA buffer were added to form the pellet for free/bound tracer separation. After a 20-h incubation, the radioactivity of the pellet was quantified using a gamma counter (ARC-1000M, Aloka, Tokyo, Japan). The range of measurement for the RIA was 3.3 to 1000 pM.

### Protein deglycosylation

LV extracts prepared for western blot and lectin blot analyses and recombinant proteins were treated with glycosidases, including sialidase, *O*-glycosidase, and PNGase F, which were all obtained from New England Biolabs (Beverly, MA). For deglycosylation, 1 μg of protein sample of interest was denatured and then treated for 4 h at 37°C with 250 U of sialidase, 40000 U of *O*-glycosidase, and/or 2500 U of PNGase F, according to the manufacturer’s protocols. After deglycosylation, samples were immediately mixed with SDS-PAGE sample buffer and then heated to stop the reaction.

### Statistical analysis

The data are presented as means ± SEM. Two groups were compared using the unpaired *t*-test with Welch’s correlation, and four groups were compared with Tukey’s honestly significant difference test (Tukey-HSD). To assess the degree of correspondence, the Pearson correlation coefficient (*r*) and *p* value were calculated for each dataset.

## Results

### Animal characteristics

Table 1 describes physiological parameters of DS rats fed HS or LS diets from 6 to 12 or to 16 weeks. At 12 weeks, HS rats exhibited severe hypertension and a decrease in body weight. The ratio of heart weight to body weight increased with hypertrophy of the LV anterior and posterior walls, and with enlargement of the LV relative to the right ventricle. Consistent with high blood pressure overload in the heart, plasma NT-proANP and NT-proBNP levels were significantly elevated in HS rats. At 16 weeks, the ejection fraction decreased, indicating an onset of a reduction in cardiac function. Continued maintenance of rats on the HS diet further decreased ejection fraction to approximately 60% of that in the age-matched LS group, increased the ratio of lung weight to body weight by 1.5-fold compared to that of the age-matched LS group, indicating pulmonary edema, and resulted in increased mortality (50% at 19 weeks). Taken together, we conclude that the 12-week HS rats had LV hypertrophy and the 16-week HS rats were in the early stage of HF with mild hypofunction.

**Table 1:** Charasteristics of DS rats at 12 and 16 weeks.

	12 weeks		16 weeks	
	LS	HS	LS	HS
*N*	6	12	6	15
BW (g)	374.1 ± 3.1	349.7 ± 5.6	404.7 ± 8.2	350.7 ± 6.2 *
HR (beat/min)	441.6 ± 11.8	448.1 ± 12.3	441.7 ± 20.8	460.4 ± 10.1
SBP (mmHg)	134.2 ± 1.5	213.9 ± 3.2 *	131.0 ± 2.3	224.1 ± 5.5 *
HW/BW (mg/g)	3.64 ± 0.05	4.72 ± 0.04 *	3.35 ± 0.05	5.15 ± 0.08 *^†^
LVW/BW (mg/g)	1.39 ± 0.07	1.89 ± 0.08 *	1.19 ± 0.07	1.83 ± 0.08 *
RVW/BW (mg/g)	0.71 ± 0.01	0.76 ± 0.02	0.65 ± 0.03	0.75 ± 0.02 *
LVAWd (mm)	1.91 ± 0.05	2.25 ± 0.06 *	2.01 ± 0.07	2.59 ± 0.06 *^†^
LVPWd (mm)	1.76 ± 0.09	2.06 ± 0.04 *	1.71 ± 0.05	2.48 ± 0.10 *^†^
EF (%)	80.8 ± 1.5	78.8 ± 0.8	81.6 ± 0.9	75.0 ± 2.1 *^†^
plasma NT-proANP (nM)	1.22 ± 0.07	3.04 ± 0.30 *	0.84 ± 0.09	4.11 ± 0.45 *
plasma NT-proBNP (pM)	33.7 ± 2.2	106.8 ± 9.2 *	37.4 ± 4.2	221.6 ± 25.8 *^†^

Data are shown as means ± SEM.

BW, body weight; HR, heart rate; SBP, systolic blood pressure; HW, heart weight; LVW (RVW), left (right) ventricular weight; LVAWd (LVPWd), left ventricular anterior (posterior) wall thickness during diastole; EF, ejection fraction.

* *p <* 0.05 (Tukey-HSD), HS group versus LS group at the same time point.

^†^ *p <* 0.05 (Tukey-HSD), HS group at 12 weeks versus that at 16 weeks.

### Identification of differentially expressed glycogenes during the progression of cardiac hypertrophy and HF

**Fig 1:**
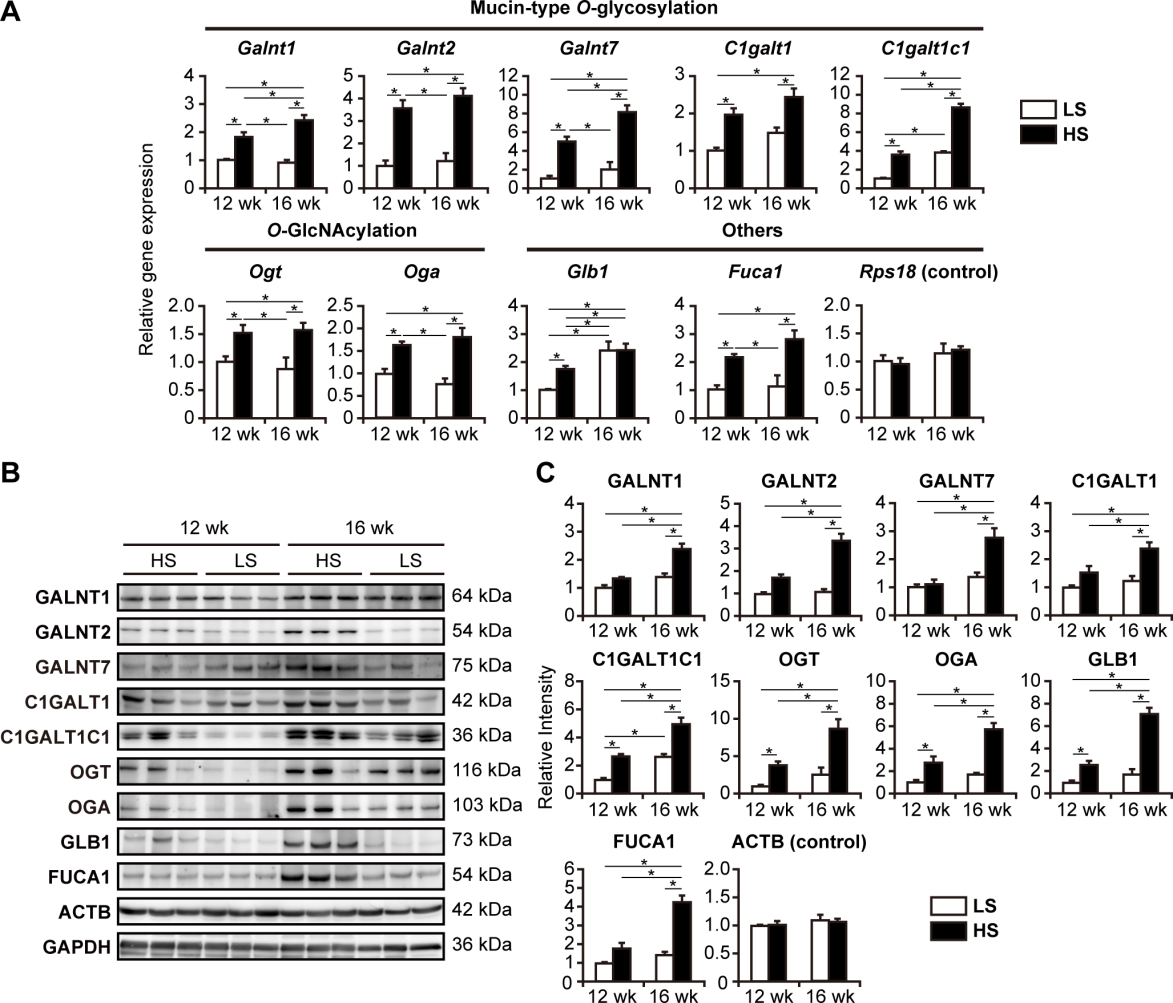
Upregulation of glycogene expression in the LV of DS hypertensive rats. (A) Relative expression levels of glycogenes selected after qPCR array in the LV of DS rats fed HS and LS diets were quantified by qPCR. *Rps18* was used as an internal control. The numbers of examined rats were n = 12 and n = 15 for the HS groups at 12 and 16 weeks, respectively; n = 6 for LS groups at each period. Expression levels were normalized to that of TATA box-binding protein (*Tbp*). (B) Protein levels of glycogenes in LV extracts were analyzed by western blotting. β-actin (ACTB) and glyceraldehyde-3-phosphate dehydrogenase (GAPDH) were used as internal controls. Representative results from three rats per group are indicated. (C) Densitometry analysis of immunoblots shown in (B). Intensity of each band was normalized to that of GAPDH. Data are presented as the fold change compared with LS rats at 12 weeks (n = 6). (A,C) *, *p <* 0.05 (Tukey-HSD).

To perform expression profiling of the genes involved in protein glycosylation (glycogenes) in the LV tissue during the progression of hypertension-induced HF, we analyzed the four treatment groups (n = 6; Table 1) using qPCR array containing 85 glycogenes (S1 Table). Gene expression levels of the 23 candidate glycogenes showing significant changes with *p* < 0.05 and/or over 2.5-fold up- or down-regulation between the HS and age-matched LS groups in the array were further verified by qPCR with gene-specific primers, which confirmed significant upregulation of nine glycogenes in HS rats compared with age-matched LS rats (Fig 1A). Among them, *Galnt1*, *Galnt2*, and *Galnt7* encoding UDP-*N*-acetyl-α-d-galactosamine:polypeptide *N*-acetylgalactosaminyltransferase 1, 2, and 7, respectively, and *C1galt1* and *C1galt1c1* encoding glycoprotein-*N*-acetylgalactosamine 3-β-galactosyltransferase (T-synthase) and its chaperone, respectively, are involved in the first two steps of the biosynthesis of mucin-type *O*-glycans. *Ogt* and *Oga* encode *O*-GlcNAc transferase and hydrolase, respectively; *Glb1* encodes β-galactosidase; and *Fuca1* encodes tissue-type AFU. Protein expression levels of these nine glycogenes in the LV of each group were compared by western blotting, which demonstrated their significant upregulation in HS rats, especially at 16 weeks (Fig 1B and 1C). These results provide direct evidence that the expression of these nine glycogenes is upregulated in DS hypertensive rats, indicating an altered state of protein glycosylation machinery in the LV during the development of cardiac hypertrophy and HF.

### Glycosylation profiles in the LV and plasma of rats with cardiac hypertrophy and HF

**Fig 2:**
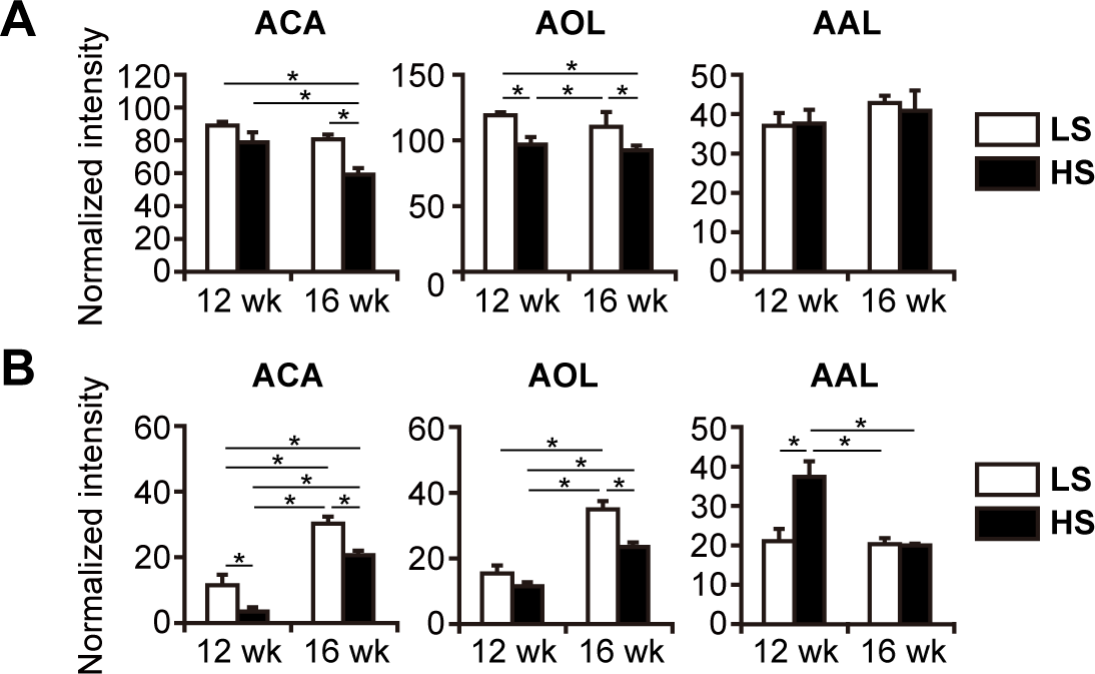
Lectin microarray analysis of LV extracts and plasma. Reactivity of fucose-binding lectins AOL and AAL, and mucin-type *O*-glycan-binding lectin ACA was analyzed in LV extracts (A) and plasma depleted of high-abundance proteins (B) of DS rats (n = 3). Entire lectin microarray datasets are shown in the S3 and S4 Tables. Data are presented as normalized intensity. *, *p <* 0.05 (Tukey-HSD).

To determine whether protein glycosylation was altered in the LV during disease progression, we compared glycoproteomes of LV extracts from the HS and LS rats by using a lectin microarray with 45 lectins; nine lectins in the HS and age-matched LS groups exhibited differential signal intensities (*p* < 0.05, S3 Table). Among them, AOL and concanavalin A (ConA) recognizing core fucose residues on *N*-glycans [22] and branched α-mannosidic structures, respectively, produced lower signals in HS rats at both time points (Table 2 and Fig 2A). In contrast, the reaction of *Aleuria aurantia* lectin (AAL) which universally recognizes α1,2/3/4/6 fucose residues on glycans [22] was not different between the HS and LS groups (Fig 2A). These results indicate that core fucose on *N*-glycans was selectively decreased in the LV glycoproteome of the HF rats. The signal from ACA recognizing mucin-type *O*-glycans, including a core 1 *O*-glycan called the T-antigen (Table 2), was decreased in the HS groups (Fig 2A), suggesting that these *O*-glycans were reduced in the LV glycoproteome of DS hypertensive rats.

**Table 2:** Summary of the lectin microarray results of LV extracts and plasma in DS rats.

Sample	Signal change	Lectin ^b^	Monosaccharide specificity ^c^	Preferred glycan structure (terminal epitope) ^c^^,^^d^
LV extract	Decrease	AOL	Fuc	Fucα1-6GlcNAc (core Fuc), Fucα1-2Galβ1-4GlcNAc (H-type 2)
		ConA	Man	High Man including Manα1-6(Manα1–3)Man
Plasma ^a^	Increase	SSA	Sia	Siaα2-6Gal/GalNAc
		TJA-I	Sia	Siaα2-6Gal/GalNAc
		Calsepa	Man	High-Man (Man_2-6_), *N*-glycans including bisecting GlcNAc
	Decrease	ABA	Gal, GlcNAc	Galβ1-3GalNAc (T-antigen), GlcNAc
		ACA	Gal	Galβ1-3GalNAc (T-antigen)
		ACG	Gal	Siaα2-3Galβ1-4GlcNAc
		STL	GlcNAc	(GlcNAcβ1–4)_n_, (GlcNAcβ1-4MurNAc)_n_ (peptidoglycan backbone)
		WGA	GlcNAc	(GlcNAcβ1–4)_n_, NeuAc

^a^ Seven high-abundance proteins were depleted before analysis.

^b^ Showing consistently changed signals both at 12 and 16 weeks. ACA, *Amaranthus caudatus* lectin; AOL, *Aspergillus oryzae* lectin; ConA, concanavalin A; SSA, *Sambucus sieboldiana* agglutinin; TJA-I, *Trichosanthes japonica* agglutinin I; Calsepa, *Calystegia sepium agglutinin*; ABA, *Agaricus bisporus* agglutinin; ACG, *Agrocybe cylindracea* galectin; STL, *Solanum tuberosum* lectin; WGA, wheat germ agglutinin.

^c^ Fuc, fucose; Man, mannose; Sia, sialic acid; Gal, galactose; GlcNAc, *N*-acetylglucosamine; GalNAc, *N*-acetylgalactosamine; MurNAc, *N*-acetylmuramic acid.

^d^ These structures are based on the review article for the lectin microarray [15].

Altered glycosylation observed in the LV of DS hypertensive rats suggested possible changes in the plasma glycoproteome, which was analyzed by the lectin microarray after depletion of high-abundance plasma proteins. More than half of the lectins tested displayed differential signals in HS rats compared with LS rats (Table 2 and S4 Table). Among them, signals of three and five lectins in the array were consistently raised and lowered, respectively, in both 12- and 16-week rats; based on lectin binding specificities, galactose and *N*-acetylglucosamine content decreased, while *N*-acetylneuraminic acid content increased, in plasma glycoproteins of HS rats (Table 2). The plasma glycoproteome of HS rats also showed decreases in the signals of *Agaricus bisporus* agglutinin (ABA) and ACA (Table 2 and Fig 2B), indicating downregulation of T-antigen levels. Additionally, proteins recognized by pan-fucose-binding lectin AAL were increased at 12 weeks, whereas proteins bound to core fucose-specific lectin AOL showed a reduction in HS rats at both time points (Fig 2B), indicating that core fucose was specifically reduced in the plasma glycoproteome regardless of the total fucose level.

### Upregulation of disialyl-T biosynthesis in the LV of DS hypertensive rats

**Fig 3:**
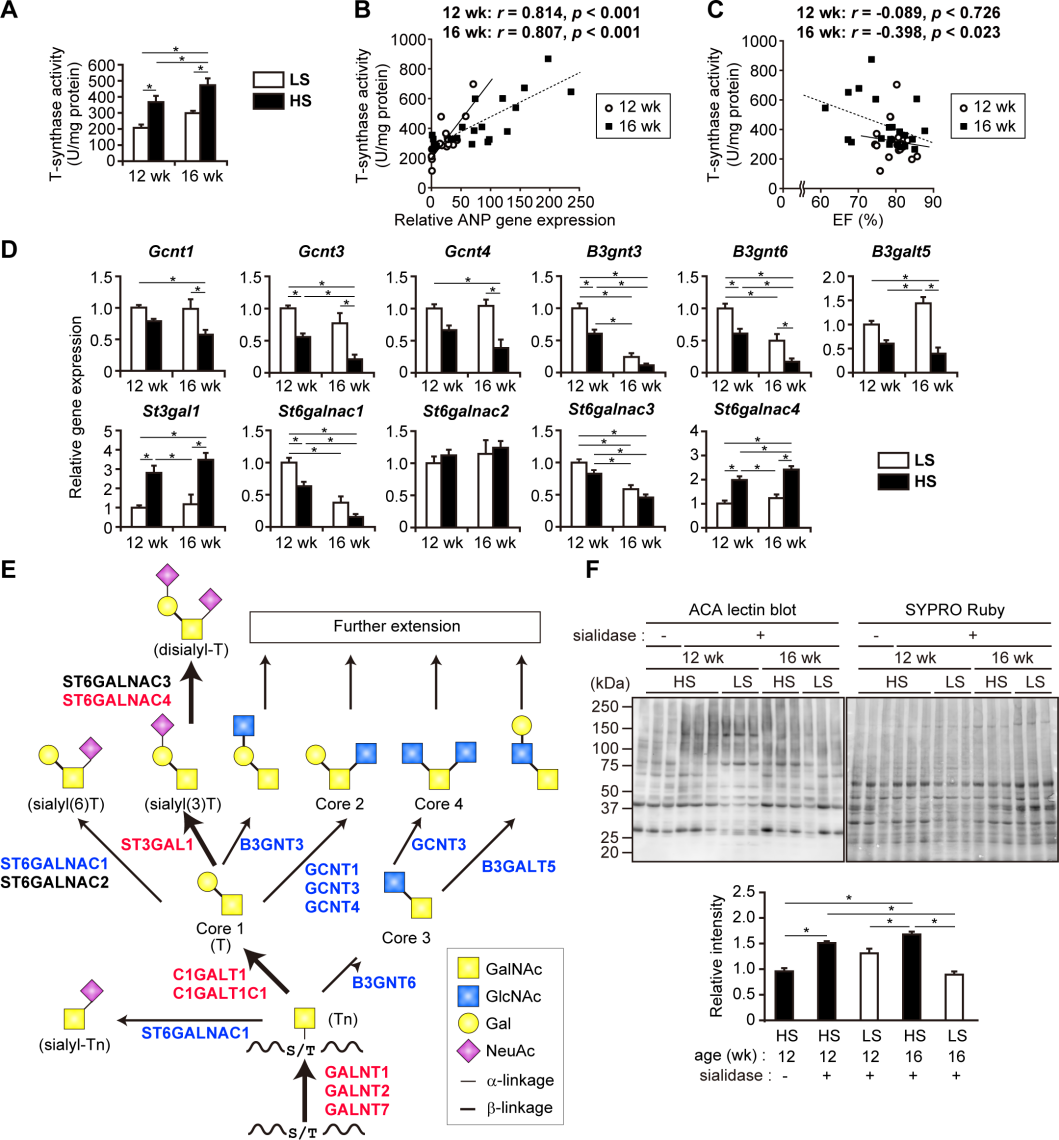
Altered mucin-type *O*-glycosylation in the LV of DS hypertensive rats. (A) T-synthase activity in LV extracts. Data were normalized to protein content. (B) Correlation of T-synthase activity with ANP gene expression. ANP gene expression level was quantified by qPCR and normalized to that of *Tbp*. Data are presented as the fold change compared with LS rats at 12 weeks. (C) Correlation of T-synthase activity with ejection fraction. (D) Relative expression levels of glycogenes involved in the early stage of mucin-type *O*-glycosylation in the LV tissues of DS rats were analyzed by qPCR and normalized to that of *Tbp*. Data are presented as the fold change compared with LS rats at 12 weeks. (E) Schematic summary of gene expression analysis data shown in (D). Examined glycosyltransferases in the mucin-type *O*-glycosylation pathway are shown in red (upregulated), blue (downregulated), or black (no change) letters. Relatively rare core structures (core 5, 6, 7, and 8) synthesized from Tn are omitted. The biosynthetic pathway of disialyl-T is upregulated, as indicated with bold arrows. GalNAc, *N*-acetylgalactosamine; GlcNAc, *N*-acetylglucosamine; Gal, galactose; NeuAc, *N*-acetylneuraminic acid. (F) Lectin blot analysis of sialidase-treated LV extracts using ACA. Representative images demonstrate ACA-reactive glycoproteins and SYPRO Ruby-stained total proteins of three individual rats in each group. Lower panel shows densitometry analysis; intensity of each band was normalized to total protein amount. Data are presented as the fold change (n = 6) compared with sialidase-untreated LV extracts of HS rats at 12 weeks. (A,D) The numbers of examined rats were n = 12 and n = 15 for the HS groups at 12 and 16 weeks, respectively; n = 6 for LS groups at each period. (A,D,F) *, *p <* 0.05 (Tukey-HSD).

Glycogene expression profiles described above indicated upregulation of the biosynthesis of mucin-type *O*-glycans in the LV of DS hypertensive rats. To determine whether this was the case, we first assessed the activity of T-synthase, which controls T-antigen biosynthesis. T-synthase activities in LV extracts obtained from HS rats were significantly higher than those from LS rats at both time points (Fig 3A). The activity showed high positive correlation (*r* > 0.8) with ANP gene expression level in the LV at both time points (Fig 3B), and a moderate, but significant, negative correlation with ejection fraction at 16 weeks (Fig 3C). These results suggest that the progression of cardiac hypertrophy and HF is accompanied by the upregulation of mucin-type *O*-glycosylation in cardiac tissues.

Next, we measured the mRNA expression levels of 11 glycogenes responsible for the early steps of *O*-glycan biosynthesis to identify which *O*-glycan structure was altered in the LV glycoproteome of the DS hypertensive rats. Nine of the tested glycogenes showed significantly different gene expression levels in the LV tissues of the HS group compared to those of the LS group at both time points (Fig 3D). The expression analysis of glycogenes involved in mucin-type *O*-glycosylation is illustrated schematically in Fig 3E, which clearly demonstrates that all the steps of disialyl-T biosynthesis were upregulated (bold arrow), whereas the conversion of main intermediates to other structures was downregulated.

We also examined whether mucin-type *O*-glycans were elevated in the LV glycoproteome of HS rats. Although the lectin microarray revealed a decrease in the amount of ACA-bound proteins, ACA lectin blot analysis detected no difference between the HS and LS rat LV tissues. Since ACA can bind to the Tn antigen, T antigen, and NeuAcα2-3Galβ1-3GalNAcα-R (sialyl(3)T), but not to sialyl(α2–6)-bound *O*-glycans, including disialyl-T [23], it was presumed that the decreased amount of ACA-bound proteins was attributed to the elevated ratio of disialyl-T to total *O*-glycans in the LV of HS rats. Therefore, we performed ACA lectin blotting for LV extracts pre-treated with sialidase to eliminate *N*-acetylneuraminic acid residues. The sialidase treatment increased the intensity of ACA-reactive bands normalized to total protein approximately 1.6-fold (Fig 3F), suggesting the presence of a significant amount of sialylated T antigens in the LV glycoproteome. The total intensity of ACA staining was notably higher in the HS group than that in the LS group after sialidase digestion (Fig 3F), indicating a significant increase in the mucin-type *O*-glycan content. Taken together, these data provide evidence that disialyl-T biosynthesis is upregulated in the LV of DS hypertensive rats.

### Upregulation of defucosylation in the LV of DS hypertensive rats

**Fig 4:**
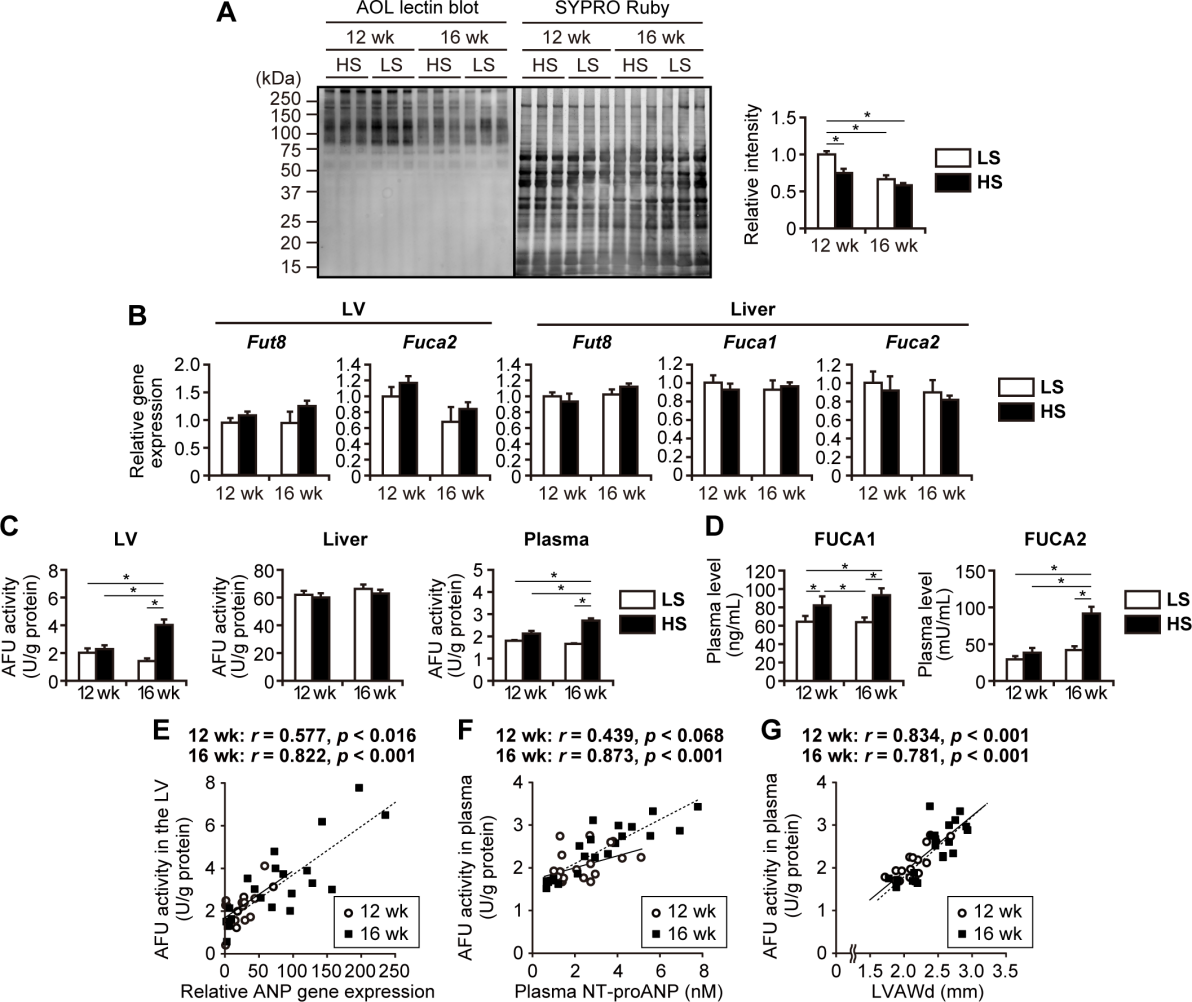
Decrease of core fucosylation on *N*-glycans in DS hypertensive rats. (A) Lectin blot analysis of LV extracts using AOL. Representative images demonstrate AOL-reactive glycoproteins and SYPRO Ruby-stained proteins of three individual rats in each group. Right panel shows densitometry analysis data; intensity of each band was normalized to total protein. Data are presented as the fold change (n = 6) compared with LS rats at 12 weeks. (B) Relative expression levels of the genes responsible for core fucosylation (*Fut8*) and defucosylation (*Fuca1* and *Fuca2*) on *N*-glycans were examined by qPCR; levels in the LV and liver were normalized to that of *Tbp* and *Actb*, respectively. Data are presented as the fold change compared with the LS group at 12 weeks. (C) AFU activity in the LV and liver extracts, and plasma. Data were normalized to protein content. (D) Plasma levels of FUCA1 and FUCA2. (E) Correlation of AFU activity in LV extracts shown in (C) with relative ANP expression shown in Fig 3B. (F,G) Correlation of plasma AFU activity shown in (C) with plasma NT-proANP concentration (*F*) and LV anterior wall thickness during diastole (LVAWd) (G). (B-G) The numbers of examined rats were n = 12 and n = 15 for HS groups at 12 and 16 weeks, respectively; n = 6 for LS groups at each period. (A-D) *, *p <* 0.05 (Tukey-HSD).

Because the expression of the *Fuca1* gene encoding AFU was elevated and there was a decrease in the signal intensity of core fucose-specific AOL in the lectin microarray, we deduced that core fucosylation of *N*-glycans is decreased in the LV tissue of DS hypertensive rats. To test this hypothesis, we compared the AOL-binding glycoprotein content in LV extracts of the HS and LS rats by AOL lectin blotting. The AOL-reactive band density relative to total protein was significantly lower in the HS group than that in the LS group at 12 weeks (Fig 4A), confirming the decrease in core fucosylation in the LV tissues of DS hypertensive rats.

To determine whether upregulation of the *Fuca1* gene occurred specifically in cardiac tissues, we analyzed *Fuca1* expression in the liver, which is the main source of plasma AFU. In contrast to the significant *Fuca1* upregulation in the LV (Fig 1), its expression in the liver was unchanged in HS rats (Fig 4B). We also tested the expression levels of *Fuca2*, which encodes an AFU isozyme FUCA2, and *Fut8*, which encodes fucosyltransferase 8, responsible for the regulation of core fucosylation; *Fuca2* and *Fut8* expression was not altered in the LV or in the liver of HS rats (Fig 4B). Moreover, AFU activity was elevated in the LV, but not in the liver, of the HS group at 16 weeks (Fig 4C). As both FUCA1 and FUCA2 are present in plasma, we also measured their plasma levels and AFU activity; plasma AFU activity and FUCA1 level were significantly elevated in HS rats at both time points, whereas FUCA2 level was elevated only at 16 weeks (Fig 4C and 4D). Collectively, these results suggest that the defucosylation of *N*-glycoproteins and the increase in plasma AFU activity were at least partly due to upregulation of the AFU activity of FUCA1 in the LV of DS hypertensive rats.

In the LV, AFU activity was positively correlated with ANP gene expression, which was moderate, but significant, at 12 weeks, and prominent at 16 weeks (Fig 4E); a similar correlation was observed with plasma NT-proANP concentration (Fig 4F), which was higher than that with plasma NT-proBNP concentration (12 weeks, *r* = 0.315; 16 weeks, *r* = 0.567). Plasma AFU activity was also positively correlated with the thickness of the LV anterior wall during diastole (Fig 4G) and the posterior wall during diastole (12 weeks, *r* = 0.534; 16 weeks, *r* = 0.726). The correlation between the LV anterior wall thickness and plasma AFU activity had a higher *r*-value than that of the LV anterior wall thickness and plasma NT-proANP level (12 weeks, *r* = 0.360; 16 weeks, *r* = 0.699). These results suggest that plasma AFU activity can be used as a biomarker for monitoring cardiac hypertrophy and HF.

### Identification of a cardiac protein with altered glycosylation

**Fig 5:**
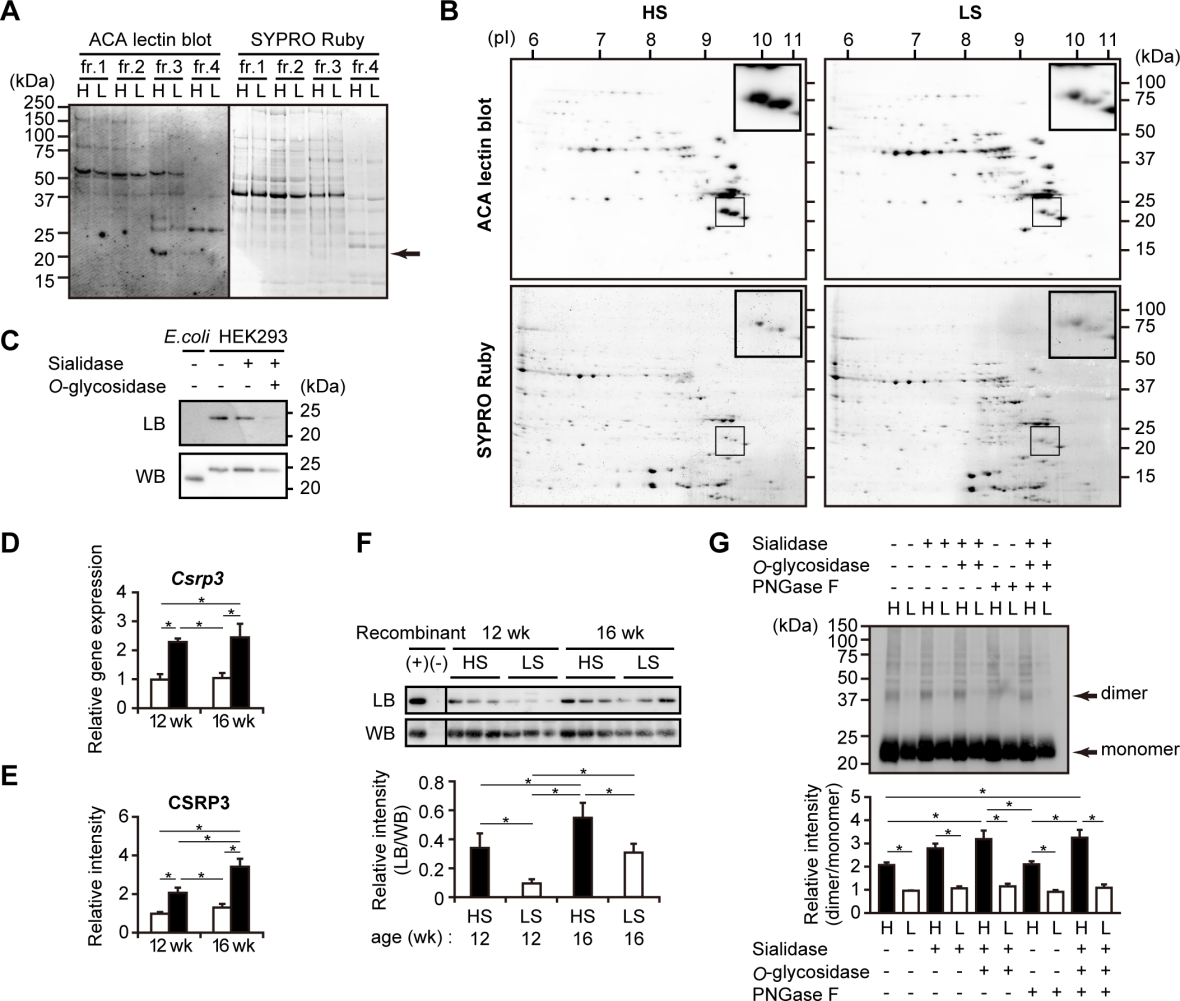
Altered *O*-glycosylation on CSRP3 in the LV of DS hypertensive rats. (A) ACA lectin blot analysis and SYPRO Ruby staining of fractions from sialidase-treated LV extracts. Arrow indicates the ACA-positive band, which is observed strongly in fraction 3 of HS (*H*) but weakly in that of the LS (*L*) group. (B) Two-dimensional PAGE images of sialidase-treated LV fraction 3. Proteins transferred to membranes were subjected to SYPRO Ruby staining, and then to ACA lectin blotting. Insets show magnified images of two spots used for protein identification. (C) Western blot (*WB*) and ACA lectin blot (*LB*) analyses of recombinant human CSRP3. Recombinant proteins expressed in *E*. *coli* (unglycosylated negative control) and in HEK293 cells (potentially glycosylated reference) were analyzed after treatment with sialidase and *O*-glycosidase. (D) Relative expression levels of *Csrp3* in the LV tissues. qPCR data were normalized to *Tbp* expression levels. The numbers of examined rats were n = 12 and n = 15 for HS groups at 12 and 16 weeks, respectively; n = 6 for LS groups at each period. (E) Protein levels of CSRP3 in LV extracts. Densitometry analysis data of western blotting are shown (n = 6). (D,E) The data are presented as the fold change compared with LS rats at 12 weeks. (F) Western blot (*WB*) and ACA lectin blot (*LB*) analyses of CSRP3 from LV extracts of DS rats. CSRP3 in LV extracts was immunoprecipitated, denatured, separated by SDS-PAGE, and analyzed. Recombinant human CSRP3 was used as an experimental control of immunoprecipitation with anti-CSRP3 antibody (+) or normal IgG (-). Lower panel shows densitometry analysis data; the intensity of each band in LB was normalized to that in WB (n = 6). (G) Effects of glycosidases on CSRP3 dimerization. LV extracts from three HS (*H*) or LS (*L*) rats at 16 weeks were treated with three glycosidases as indicated and then analyzed by western blotting for CSRP3. Arrows indicate the bands corresponding to monomers and dimers. Lower panels show densitometry analysis from five experiments; dimer/monomer ratios are presented as the fold change compared with LS rats without glycosidase treatment. (D-G) *, *p <* 0.05 (Tukey-HSD). In (G), statistical comparison of HS and LS groups in the same condition and that of HS groups in five conditions are indicated.

Since altered amount of ACA-bound proteins was observed (Fig 3F), we conducted differential proteomic analysis targeting ACA-bound proteins to address which cardiac proteins were actually subject to altered *O*-glycosylation in DS hypertensive rats. Proteins in LV extracts after sialidase treatment were divided into four fractions according to solubility; the solubility of proteins was reduced from fraction 1 to fraction 4. A lectin blot analysis of proteins separated by SDS-PAGE revealed that an ACA-positive band of approximately 21 kDa in fraction 3 showed markedly higher intensity in the HS group than in the LS group (Fig 5A). The proteins in fraction 3 were further analyzed by two-dimensional PAGE; the two closely located spots of approximately 21 kDa in the HS group exhibited higher ratios of ACA signal intensity to total protein amount than those observed in the LS group (Fig 5B). Mass spectrometry analysis revealed that both of the spots were CSRP3 by identification of 5–6 tryptic peptides, which was further confirmed by western blot analysis using an antibody against CSRP3 (S1 Fig). To estimate whether CSRP3 was *O*-glycosylated, recombinant human CSRP3 proteins expressed in HEK293 cells and in *E*. *coli* were compared in terms of affinity for ACA; HEK293 cells have a set of glycosylation machinery, whereas *E*. *coli* does not. Recombinant CSRP3 of HEK293 origin was ACA-positive, but CSRP3 of *E*. *coli* origin was not (Fig 5C). Moreover, ACA-binding activity of HEK293-derived CSRP3 was abolished by treatment with both sialidase and *O*-glycosidase, but not with sialidase alone (Fig 5C). These results indicate that CSRP3 is *O*-glycosylated. In the LV of DS hypertensive rats, mRNA and protein levels of CSRP3 were upregulated (Fig 5D and 5E), which was accompanied by altered *O*-glycosylation (Fig 5F). Western blot analysis showed that CSRP3 dimerized in unheated/non-reducing conditions (Fig 5G). In the HS group, the dimer to monomer ratio was increased by sialidase and *O*-glycosidase treatment, but not with PNGase F treatment (Fig 5G), suggesting the involvement of sialic acid and *O*-glycans in CSRP3 oligomerization.

## Discussion

In the present study, we analyzed changes in protein glycosylation machinery and the glycoproteome in the LV and plasma of DS hypertensive rats, a disease model of hypertension-induced cardiac hypertrophy and HF. Our analysis revealed the upregulation of β-galactosidase and *O*-GlcNAcylation-related genes known to be involved in cellular senescence [24] and cardiac diseases in humans and animal models [25,26]. Moreover, we identified significant alternations in the content of two glycan structures in the LV glycoproteome: an increase in mucin-type *O*-glycans, especially disialyl-T, and a decrease in core fucose on *N*-glycans. These differential glycosylation patterns observed in the LV of hypertrophic and failing hearts indicate their important roles in the pathophysiology of HF.

Mucin-type *O*-glycosylation is initiated by a large family of polypeptide *N*-acetylgalactosaminyltransferases [27], including GALNT1, GALNT2, and GALNT7, which were found to be upregulated in the LV of HS rats (Figs 1 and 3E). GALNT1 and GALNT2 are responsible for the first step of *O*-glycosylation on non-glycosylated polypeptides [28], whereas GLANT7 catalyzes additional glycosylation on already *O*-glycosylated polypeptides [29]. Considering the substrate specificity of these three transferases, our data suggest that the density of *O*-glycans on *O*-glycosylated proteins, as well as initiation of *O*-glycosylation on non-glycosylated proteins, are elevated in the LV of DS hypertensive rats. We also observed upregulated expression of C1GALT1 and its molecular chaperone C1GALT1C1, along with increased T-synthase activity in the LV of DS hypertensive rats, indicating upregulation of T-antigen synthesis, the rate-limiting step of mucin-type *O*-glycosylation. C1GALT1C1 was more significantly upregulated than C1GALT1, consistent with the key regulatory role of this chaperone in maintaining correct protein *O*-glycosylation [30]. An increase in ACA lectin binding for sialidase-treated LV extracts of HS rats was observed for proteins of a broad molecular weight range (Fig 3E), suggesting that the upregulation of *O*-glycosylation is not limited to specific proteins but common to a variety of proteins in the LV proteome.

Aberrations in mucin-type *O*-glycosylation are known to be associated with various pathophysiological conditions. *Galnt1*-knockout mice exhibit impaired cardiac valvulogenesis and compromised cardiac function that mimics human congenital heart disease, and is associated with reduced *O*-glycosylation on multiple proteins in heart valve tissues [31]. Another example is the significant upregulation of *C1galt1c1* expression accompanied by an increase in T-antigen in the liver of mice with CCl_4_-induced hepatic fibrosis [32]. Because fibrosis is an indication of remodeling in the hypertrophied LV [33], which is one of characteristic consequences of Dahl hypertensive rats [34], *C1galt1c1* expression may be regulated by similar signaling pathways in both the liver and heart. In contrast, we observed an increase in disialyl-T in the LV of the hypertrophied and failing heart, while sialyl(3)T was elevated in the fibrotic liver [32]. These facts indicate that heart and liver have intrinsically distinct regulatory mechanisms for *O*-glycosylation machinery.

By proteomic analysis, we identified CSRP3 as a candidate protein to elucidate the relationship between aberrant *O*-glycosylation and cardiac dysfunction in DS hypertensive rats. CSRP3, an *O*-glycoprotein of 21kDa, is a key regulator of cardiac muscle physiology and pathophysiology, functioning as a stress sensor and a mediator of stretch signaling [35]. After sialidase treatment, there was a dramatic difference in CSRP3 *O*-glycosylation detected by ACA-binding in LV extracts of HS rats and LS rats (Fig 5). CSRP3 is present as a monomer and oligomer in the nucleus and cytoplasm, respectively [36], and nucleocytoplasmic shuttling is required for cardiomyocyte remodeling in response to hypertrophic stimuli [37]. In this context, our data raise a possibility that aberrant *O*-glycosylation on CSRP3 alters its cellular localization and function by changing the monomer to oligomer ratio. CSRP3 has been reported to exert complex and diverse effects by interacting with multiple proteins [35]. Using the NetOglyc prediction [38], six potential mucin-type *O*-glycosylation sites on the rat and human CSRP3 sequences are localized at the inter-LIM region, which can serve as the binding site for its partner proteins [39]. Thus, the *O*-glycosylation state of CSRP3 may affect interactions with its transcriptional regulators, including calcineurin, a mechanotransduction mediator in cardiomyocytes [40]. Taken together, the alteration of glycosylation states of proteins certainly has the potential to regulate cellular signals from mechanical stress and hypertrophy, as well as cardiomyocyte remodeling.

Our observations indicate that disturbance of the coordinated regulation of the glycosylation machinery in the heart tissue results in the upregulated production of disialyl-T and the suppression of conversion into the other glycan structures (Fig 3). Although the mechanisms underlying regulation of glycogene expression remain to be clarified, several studies have reported that micro RNAs [41,42], such as miR-30b/d targeting both Galnt1 and Galnt7 [43] and epigenetic modifications, contribute to the regulation of glycogene expression in cancer cells and leukocytes [44,45]. These hierarchical regulatory systems executing glycosylation processes may strongly influence the pathogenesis of cardiac diseases. The activity of T-synthase, the only enzyme catalyzing the rate-limiting reaction in this glycosylation pathway, exhibited significant correlation with ANP gene expression (Fig 3B), suggesting that the regulatory mechanisms underlying mucin-type *O*-glycosylation may be associated with the progression of cardiac hypertrophy. On the other hand, alterations in glucose metabolism may affect the glycosylation machinery and glycoproteome in the heart tissue, since it has been reported that DS rats fed high-salt diets exhibit hypertension-induced insulin resistance [46].

We originally pursued a study to elucidate the mechanism of *O*-glycosylation of human proBNP [47]. Our present study demonstrated that the upregulation of mucin-type *O*-glycosylation correlated with the progression of cardiac hypertrophy and HF; this may contribute to the increase in sialylated *O*-glycan at Thr71 close to the processing site, resulting in the inhibition of proBNP conversion to bioactive BNP-32 by hindering the binding of a processing protease, furin [48]. A similar mechanism was observed in the conversion of angiopoietin-like protein 3; site-specific *O*-glycosylation close to the furin cleavage motif prevents processing to the mature protein [49]. Because GALNT2 is the enzyme responsible for *O*-glycosylation of angiopoietin-like protein 3 [49], the upregulation of GALNT2 expression in stressed cardiomyocytes may contribute to the aberrant *O*-glycosylation of proBNP.

Here, we consistently detected the upregulation of *Fuca1* encoding tissue-type AFU, and the reduction of core fucosylation on *N*-glycoproteins in the LV of DS hypertensive rats. Decreased core fucosylation was also observed in tumor tissues and serum of gastric cancer patients [50]. In *Fut8*-deficient mice, defucosylation of *N*-glycans on transforming growth factor β1 receptor and epidermal growth factor receptor has been identified as a key factor deregulating the signaling pathways [51,52]. Because the signaling of these two growth factor receptors plays a pivotal role in tissue remodeling in cardiac hypertrophy and failing heart [53,54], defucosylation of these receptors may contribute to remodeling in heart disease.

The degree of core fucosylation related to cardiac remodeling can be easily detected by core fucose-specific lectins; therefore, core fucosylation can be considered as a candidate biomarker of cardiac pathology. A well-known example is the detection of fucosylated α-fetoprotein, which is useful in the diagnosis of hepatocellular carcinoma [55]. The decrease in AOL-specific signal observed in plasma and the LV of hypertensive rats indicates that defucosylated glycoproteins are potential biomarkers for cardiac hypertrophy and HF. Furthermore, our study shows that differential expression of glycogenes may be another possible biomarker candidate for cardiac hypertrophy and HF, although it is difficult to measure the tissue expression levels of glycogenes. Instead, our data demonstrate the advantage of measuring plasma AFU activity as a biomarker for cardiac hypertrophy; it is easily measurable, significantly elevated in hypertensive rats, and exhibited significant correlation with LV wall thickness (Fig 4). Although our results do not exclude the possibility that other tissues, such as the liver and blood vessels, may contribute to the elevation of plasma AFU protein and activity in HS rats, this activity is a practical biomarker candidate for the diagnosis of cardiac hypertrophy, which may have slightly different properties from natriuretic peptides.

The present study had several limitations. The present study utilized the LV tissue lysates, which contain various types of cells such as cardiomyocytes, fibroblasts, and vascular wall cells. Thus, further study is required to identify which type of cells serves altered glycosylation phenotype in the LV of hypertensive rats. In addition, altered glycosylation was monitored by using lectins, which exhibits a wide range of glycan specificity; the next crucial step is to identify glycoproteins and their glycan structures responsible for exhibiting differences in the lectin blotting between heart tissues of HS and LS rats. Characterization and molecular identification of altered glycosylation coupled with identification of carrier glycoproteins by glycomic and proteomic approaches mainly using mass spectrometers are essential to elucidate the contribution of alteration of glycan structures to the pathophysiology of HF.

In conclusion, we identified specific glycan structures showing changes during the development of cardiac hypertrophy and HF in the rat model. Our study also provides insights into the molecular mechanism of aberrant glycosylation in cardiac hypertrophy, particularly by highlighting the significance of *O*-glycosylation changes on CSRP3. The glycosylation machinery, including the glycogenes tested here, is conserved in human and rats, indicating that similar pathophysiological situations may occur in humans. The present study suggests that the plasma glycoproteome, a mixture of proteins secreted from various tissues, reflects dynamic changes occurring in glycoproteins secreted from the heart and is certainly a very promising source of circulating biomarkers for cardiac diseases. Elucidation of the pathophysiological significance of aberrant glycosylation promotes our understanding of the molecular mechanisms underlying HF progression, and accelerates the discovery of reliable diagnostic biomarkers and identification of therapeutic targets.

## Supporting Information

S1 Fig **Western blot (*WB*) and lectin blot (*LB*) analyses of SDS-PAGE (A) and two-dimensional PAGE (B) gels.** Membranes with transferred proteins from each gel were sequentially subjected to SYPRO Ruby staining, LB using ACA, and WB using an anti-CSRP3 antibody.
(TIF)

S1 Table Glycogenes tested by the qPCR array. (XLS)

S2 Table Primers used for qPCR. *Actb*, β-actin; ANP, atrial natriuretic peptide; *Rps18*, ribosomal protein S18; *Tbp*, TATA box binding protein.
(XLS)

S3 Table Lectin array data of LV extracts from DS rats. The data are presented as means ± SEM (n = 3). *p < 0.05 (unpaired *t*-test with Welch's correlation), HS group vs LS group at the same time point.
(XLS)

S4 Table Lectin array data of plasma from DS rats. Seven high-abundance proteins were depleted from plasma specimen before analysis. The data are presented as means ± SEM (n = 3). *p < 0.05 (unpaired *t*-test with Welch's correlation), HS group vs LS group at the same time point.
(XLS)
