# Supplementary figures and images for: Aberrant Glycosylation in the Left Ventricle and Plasma of Rats with Cardiac Hypertrophy and Heart Failure
Source: PLoS One. 2016 Jun 9;11(6):e0150210. doi: 10.1371/journal.pone.0150210 (PMC4900630; doi:10.1371/journal.pone.0150210)

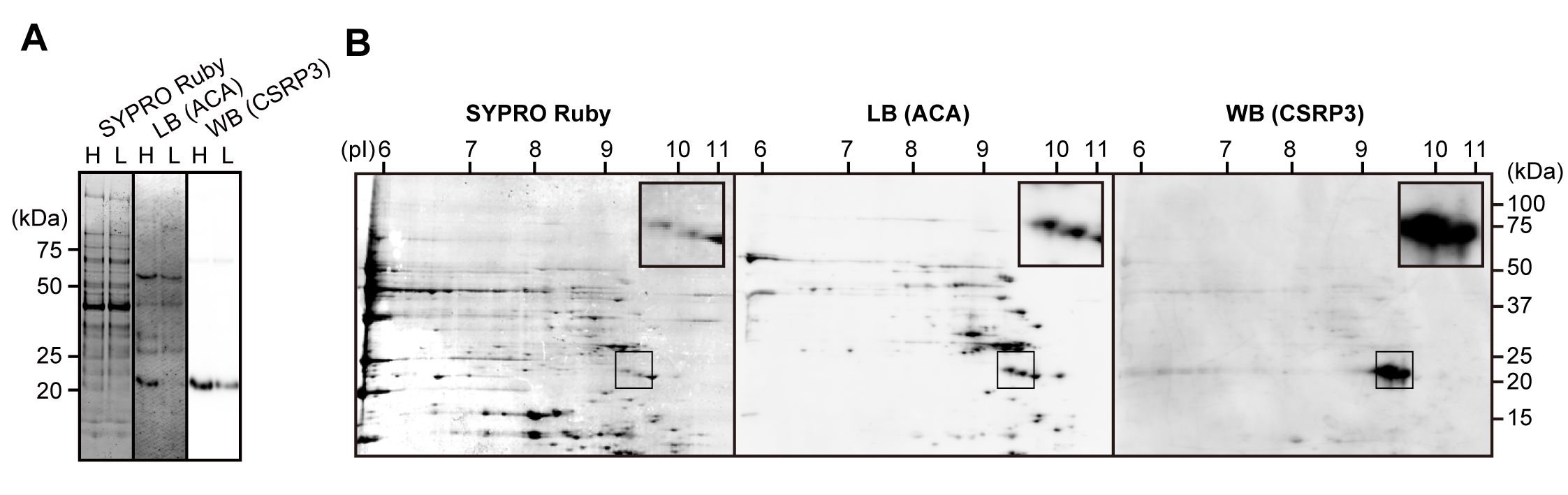

Supplement: S1 Fig — Western blot (WB) and lectin blot (LB) analyses of SDS-PAGE (A) and two-dimensional PAGE (B) gels. Membranes with transferred proteins from each gel were sequentially subjected to SYPRO Ruby staining, LB using ACA, and WB using an anti-CSRP3 antibody. (TIF) [file pone.0150210.s001.tif]
